# Development and Evaluation of Multi-Module Retinal Devices for Artificial Vision Applications

**DOI:** 10.3390/mi16050580

**Published:** 2025-05-15

**Authors:** Kuang-Chih Tso, Yoshinori Sunaga, Yuki Nakanishi, Yasuo Terasawa, Makito Haruta, Kiyotaka Sasagawa, Jun Ohta

**Affiliations:** 1 Institute for Research Initiatives, Nara Institute of Science and Technology, Ikoma 6300192, Japan; 2 Division of Materials Science, Graduate School of Science and Technology, Nara Institute of Science and Technology, Ikoma 6300192, Japan; 3 NIDEK Co., Ltd., Gamagori 4430036, Japan; 4 Department of Opto-Electronic System Engineering, Faculty of Science and Engineering, Chitose Institute of Science and Technology, Chitose 0668655, Japan; 5 Medilux Research Center, Graduate School of Science and Technology, Nara Institute of Science and Technology, Ikoma 6300192, Japan

**Keywords:** artificial retina device, suprachoroidal transretinal stimulation (STS), smart CMOS system

## Abstract

Artificial retinal devices require a high-density electrode array and mechanical flexibility to effectively stimulate retinal cells. However, designing such devices presents significant challenges, including the need to conform to the curvature of the eyeball and cover a large area using a single platform. To address these issues, we developed a parylene-based multi-module retinal device (MMRD) integrating a complementary metal-oxide semiconductor (CMOS) system. The proposed device is designed for suprachoroidal transretinal stimulation, with each module comprising a parylene-C thin-film substrate, a CMOS chip, and a ceramic substrate housing seven platinum electrodes. The smart CMOS system significantly reduces wiring complexity, enhancing the device’s practicality. To improve fabrication reliability, we optimized the encapsulation process, introduced multiple silane coupling modifications, and utilized polyvinyl alcohol (PVA) for easier detachment in flip-chip bonding. This study demonstrates the fabrication and evaluation of the MMRD through in vitro and in vivo experiments. The device successfully generated the expected current stimulation waveforms in both settings, highlighting its potential as a promising candidate for future artificial vision applications.

## 1. Introduction

Recent years have witnessed a marked increase in the incidence of retinal neuron degeneration, such as retinitis pigmentosa and age-related macular degeneration [1,2]. These conditions are particularly challenging to diagnose and treat, prompting a surge in research focused on integrating electronics with neural engineering to develop effective therapeutic solutions. For several decades, implantable bioelectronic devices have been designed to replace degenerated retinal neurons such as photoreceptor cells with functional alternatives [3,4,5,6,7,8,9,10,11,12,13]. The fundamental therapeutic mechanism of these devices involves the application of electrical charges via bioelectrodes to stimulate neurons. These charges modulate local potentials across neuronal membranes, and once the potential reaches the threshold, an action potential of neurons is initiated. Once stimulated, these neurons transmit modified electrical signals throughout the neural network, potentially restoring neural functions [3,4,5,6,7,8,9,10,11,12,13].

Among the various bioelectronic interventions, retinal implants have been developed, with notable prototype examples including the Alpha IMS (Retina Implant AG, Germany) [3,4,13], the Argus II (Second Sight Inc., Sylmar, USA) [5,6,13], and the Artificial Vision Project led by Bionic Vision (Melbourne, Australia) [8,14,15], while a Japanese group led by Osaka University and NIDEK Co., Ltd. [7,9], as well as several teams, such as Stanford University and the Pixium Vision group, have been working on a cutting-edge retinal implant [16,17,18,19]. These implants are placed in different retinal regions, which can be categorized into three main locations: epiretinal, subretinal, and suprachoroidal. The Argus II and Alpha IMS devices are positioned in the epiretinal and subretinal regions, respectively. However, these implantation procedures are highly invasive and pose significant risks, such as the need for anchoring to secure the device to retinal tissue and potential obstruction of the nutritional pathway. In contrast, suprachoroidal transretinal stimulation (STS) has emerged as a safer alternative, as demonstrated in clinical trials [20,21,22]. Moreover, this less invasive approach has successfully validated the feasibility of retinal implants utilizing the STS method.

In previous studies, we developed an STS device using platinum (Pt) and sputtered iridium oxide (IrO_x_) as stimulation electrodes [23,24,25,26]. Additionally, we reported a single-module ceramic retinal device (employing Pt electrodes) and introduced a facile fabrication process for high-performance stimulation electrodes (through chemical bath deposition), enabling the integration of IrO_x_ electrodes into our retinal device [25]. Using this single-module retinal device, we successfully confirmed the evoked potentials induced by electrical stimulation in animal experiments [26]. However, to achieve high-resolution artificial vision devices, multiple modules and a greater number of stimulation electrodes are necessary.

To address the requirements of multi-module retinal devices (MMRDs), we developed a new, improved process for a flexible, multi-module retinal device. This device incorporates multiple modules (biocompatible ceramic substrates), a biocompatible parylene-C (PaC) flexible substrate, well-encapsulated titanium (Ti)/gold (Au) thin-film wiring, and platinum (Pt) electrodes. In this study, we present the improved fabrication process for achieving a multi-module configuration, highlighting advancements in device structures, material selection, and a device assembly. Furthermore, we evaluate the performance of MMRDs and conduct animal experiments to assess neural responses by electrical evoked potential (EEP) and its feasibility for retinal implantation. These findings contribute to the advancement of next-generation STS retinal prostheses offering improved adaptability and reduced surgical risks.

## 2. Materials and Methods

### 2.1. Structure of the Flexible MMRD

**Figure 1:**
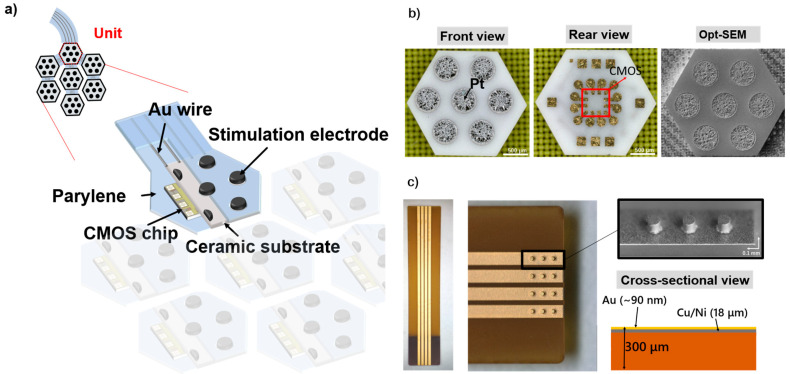
(**a**) Structure of the flexible MMRD. (**b**) Optical images of the ceramic substrate, showing front, rear, and Opt-SEM views (from left to right). (**c**) The optical and Opt-SEM images of the FPC substrate.

Herein, we propose a flexible MMRD composed of robust ceramic substrates (Nikko Ceramics Inc., Hakusan, Japan), smart complementary metal-oxide semiconductor (CMOS) chips, and a primary supported PaC substrate. Each ceramic unit was connected using Au/Ti wires, as illustrated in Figure 1a. The ceramic substrates measured 2.30 mm × 2.65 mm. The front side of each ceramic substrate housed seven Pt electrodes (0.8 mm pitch), with the corresponding Au interconnects/pads for each electrode and CMOS chip designed on the rear side (Figure 1b). On the rear view, the pads were labeled VDD, GND, C1, and C2 to facilitate connections between ceramic units (Au/Ti wiring) and CMOS chip bonding. Figure 1b also depicts the optical shadow effect mode (Opt-SEM, Keyence, Japan) images of the ceramic substrate, featuring 500 µm Pt electrodes (with no cracks and voids). Moreover, a flexible printed circuit (FPC) substrate with Au bumps is used to manage power and electrical signal control for the flexible MMRD. The thickness of the Au/CuNi wires for the FPC substrate is 90 nm/18 µm with Au bumps, as shown in Figure 1c.

**Figure 2:**
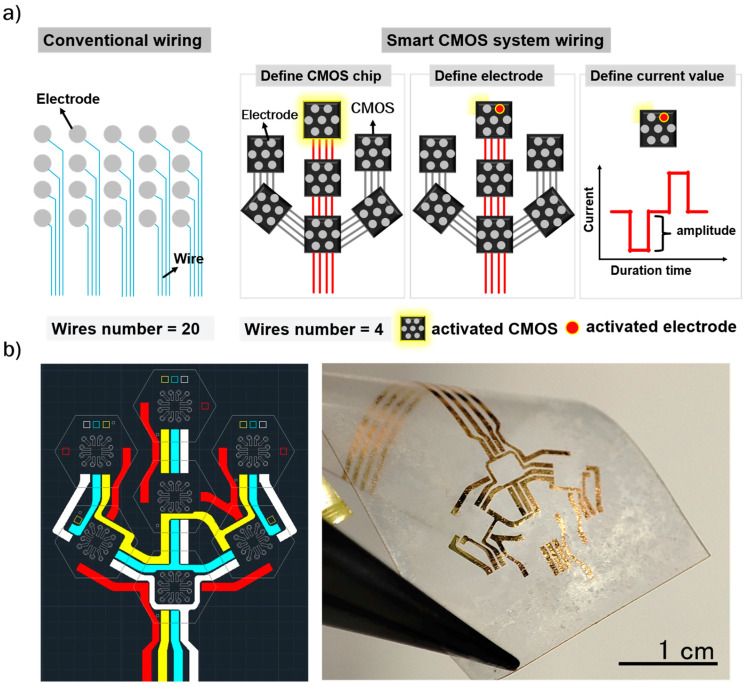
(**a**) Illustration of the wiring method using conventional and smart CMOS systems. (**b**) Wiring design and image of the wires for the flexible MMRD.

Wiring design is a critical aspect of bioelectronics, especially regarding the spacing and number of wires needed, which can become a bottleneck in high-resolution signal recording and neural stimulation. For instance, connecting 20 electrodes typically requires 20 wires, creating considerable difficulties both in wiring design and when scaling up to larger numbers of electrodes (as illustrated in Figure 2a). These challenges can limit the use of bioelectronics for complex neural network studies or high-resolution neural stimulation. Therefore, to address these difficulties, our study introduces a smart CMOS system designed to simplify wiring engineering. The CMOS chip comprises three primary functional circuits: a control circuit, a current generation circuit, and an electrode-switching circuit. Chip operation is regulated by two external control signals (Control Signal 1 and Control Signal 2), which coordinate with the internal control and current generation circuits to determine the stimulation current. Each CMOS chip is fabricated using a 0.35 µm standard CMOS process and is subsequently diced into individual units with dimensions of 500 × 500 µm^2^. The chip operates with a 5 V DC power supply and is designed to generate biphasic, cathodic-first pulse currents for electrical stimulation. The output current is delivered to the selected electrode through the integrated electrode-switching circuit. Signal control and power delivery are achieved through a four-wire interface, consisting of one power line, two control lines, and one ground line. Each chip includes a dedicated Chip ID circuit to enable independent addressing and precise operation within the multi-module system. This configuration significantly reduces wiring complexity, supports modular scalability, and minimizes signal interference between channels. The detailed specifications and operation mechanisms of the CMOS chip are provided in our previous work [24,25,26]. Notably, our proposed flexible MMRD, which incorporates the smart CMOS system, requires only four wires to operate 49 electrodes. This represents a significant reduction in wiring complexity, from 49 wires to four wires (Figure 2b). Additionally, the CMOS system allows precise control of neural simulation by manipulating each chip and electrode via their respective identification markers. Therefore, the proposed flexible MMRD with the smart CMOS system stands out as a promising retinal device.

### 2.2. Fabrication of the Flexible MMRD

**Figure 3:**
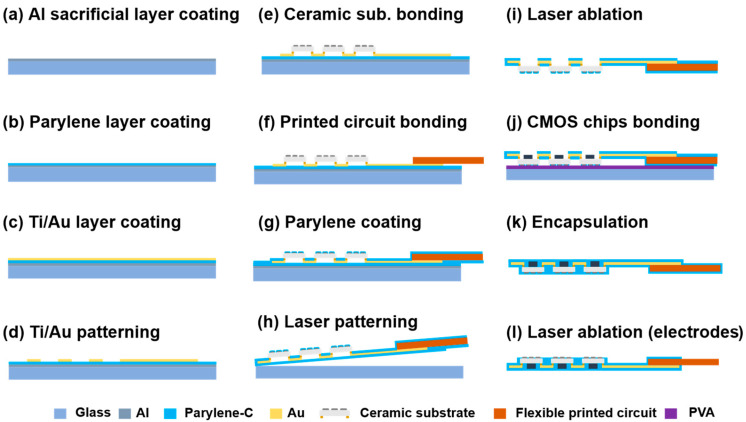
Flowchart illustrating the flexible MMRD fabrication process.

Figure 3 presents the fabrication flowchart of the flexible MMRD. A glass substrate served as the support for the device fabrication process. The glass substrate was cleaned by immersion in acetone and isopropanol for 10 min. This was followed by plasma treatment (50 W) using oxygen gas (O_2_) at a flow rate of 10 mL/min for 1 min to remove surface contaminants. Subsequently, a sacrificial aluminum (Al) layer (100 nm) was deposited on the glass substrate via evaporation deposition (VPC-260F, ULVAC). To create the primary supporting substrate for the device, a 5 µm thick layer of PaC was deposited on top of this Al layer via chemical vapor deposition (PDS 2010, Specialty Coating Systems, Indianapolis, USA). Next, a metal layer (50 nm Ti/100 nm Au) was deposited by sputtering to serve as the wiring for the device. The Ti/Au metal layer was then patterned using photolithography (Mikasa-M30, Minato, Japan) and chemical etching (the etching solutions are listed in Table S1). The ceramic substrates were mounted on the Ti/Au wires through a flip-chip bonding process (M-90, HiSol, Taito, Japan). The ceramic and FPC substrates were connected to the Ti/Au wires using an anisotropic conductive paste (TAP0402E, Kyocera, Kyoto, Japan) under 11 N of pressure at 130 °C for 60 s. Following this, a second PaC layer was deposited to encapsulate the Ti/Au wires. Prior to PaC encapsulation, both the Au interconnects and the initial PaC layer were treated with a silane solution at 25 °C for 15 min to enhance adhesion between the two PaC layers. After the second PaC layer was applied, the device was cut by UV laser ablation using a Q-switched Nd:YAG (neodymium-doped yttrium aluminum garnet) laser at a wavelength of 266 nm, which was utilized as a lift-off process to detach the device from the supported glass substrate. This was followed by a lift-off process in an alkaline solution (1 M sodium hydroxide) to remove the sacrificial Al layer, as shown in Figure 3h. For the CMOS flip-chip process, the PaC layer covering pads at the ceramic substrate for CMOS chip bonding was selectively removed by the UV laser combined with O_2_ plasma treatment. Before the flip-chip bonding process for CMOS chips, the sample was fixed on glass supporting substrate using polyvinyl alcohol (PVA). The flip-chip bonding was then performed under the same conditions as those used for the ceramic substrates. Finally, after detaching the sample from the glass substrate by dissolving the PVA in water, a second silane coupling modification process was performed. Subsequently, a third PaC layer was deposited for final encapsulation, and UV laser exposure was used to reveal the electrodes.

### 2.3. Operating System of the MMRD

**Figure 4:**
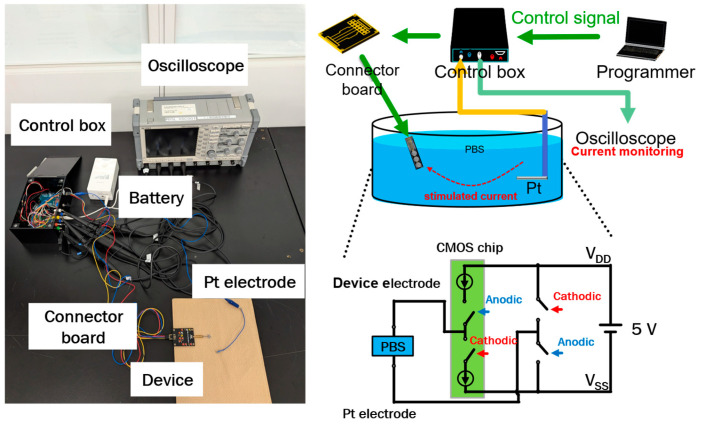
Photograph and illustration of the operating system setup for the flexible MMRD.

The device was controlled using a signal control box equipped with a floating power supply system, allowing for flexibility in the voltage supply. The counter electrode was connected to a voltage source (either V_DD_ or V_SS_), while a switchable circuit regulated whether the cathodic or anodic current was directed to the stimulating electrode. A block diagram and a photograph of the operational setup are displayed in Figure 4, with further details provided in our previous publications [25,26]. The experimental configuration, depicted in Figure 4, included a personal computer (PC) programmer, a signal control box, a receiver multiplexer, a Pt counter electrode, and an oscilloscope. The PC programmer and signal control box were employed to configure different current values for the targeted electrodes. The device was connected to a connector board to process four input signals. The connector board consisted of four signal sources to receive two power sources (V_DD_ and V_SS_), and two control sources from the control box. The experiment was conducted in a phosphate-buffered solution (PBS, Wako Pure Chemical Industries, Osaka, Japan) at room temperature, with both the device and the Pt counter electrode immersed to simulate a physiological environment. To assess the output current responses, a bipolar stimulation waveform was operated as intended (ranging from 250 to 1000 μA). The output currents were observed by a 20 MS/s isolated recorder oscilloscope (GR-7000, Keyence, Osaka, Japan).

### 2.4. Animal Experiments

#### 2.4.1. Rat Preparation

Adult Long–Evans rats (Japan SLC) (*n* = 2) were subjected to a 12 h light/12 h dark cycle with ad libitum access to standard food and water. All rats were anesthetized prior to surgery, and acute electrophysiological recordings were conducted following intraperitoneal injection of urethane (1.75 g/kg). Sevoflurane inhalation was used for initial anesthesia. During surgery and experimentation, the rats were secured in a stereotaxic apparatus (Narishige, Setagaya, Japan). The basic surgical procedure followed a previously established protocol [27].

#### 2.4.2. Rabbit Preparation

An adult rabbit (Kbl:JW, Kitayama Labes Co., Ltd., Inashi, Japan) (*n* = 1) was subjected to a 12 h light/12 h dark cycle, with ad libitum access to standard food and water. The animal was anesthetized for surgery and acute electrophysiological recordings (via sevoflurane inhalation). Initial anesthesia was induced through an intramuscular injection of a mixture of ketamine, xylazine, and butorphanol. During surgery and experimentation, the rabbit was positioned in a stereotaxic apparatus (S4329, Narishige, Setagaya, Japan). An intrascleral pocket was created in the eyeball, into which the stimulus head of the fabricated device was inserted. The counter electrode was placed subcutaneously into the left front leg.

### 2.5. Electrophysiological Recordings

We measured the electrically evoked potential (EEP) induced by electrical stimulation of the rat retina. A tungsten (W) microelectrode (resistance: 0.5 MΩ; diameter: 5.0 µm (uninsulated part); Unique Medical, Tokyo, Japan) was set at a depth of 250 µm from the surface of a sclera (SC) using a micromanipulator (MMO-203; Narishige). EEPs were amplified using a differential amplifier (DP-301, Warner Instruments, Holliston, MA, USA) (amplification: 100 times; electrical stimulation: 1000 times; band-pass filter: 10 Hz–3 kHz) and then digitally sampled. The collected data were averaged using PowerLab (PowerLab 2/26 (ML 826); ADInstruments, Dunedin, New Zealand) and data acquisition software (LabChart, ADInstruments). The sampling frequency was set at 10 kHz for 30 electrical stimulations. All subsequent recordings were taken in this position.

## 3. Results

### 3.1. Imaging of the Fabricated Device

**Figure 5:**
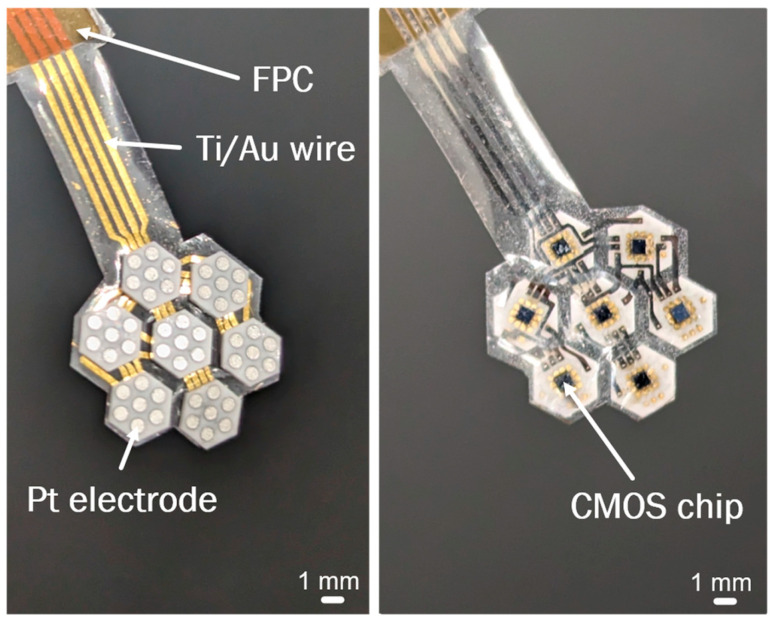
Photograph of the flexible MMRD.

The completed flexible MMRD, with Ti/Au wires embedded in the PaC, is displayed in Figure 5.

#### In Vitro Experiment in the PBS

**Figure 6:**
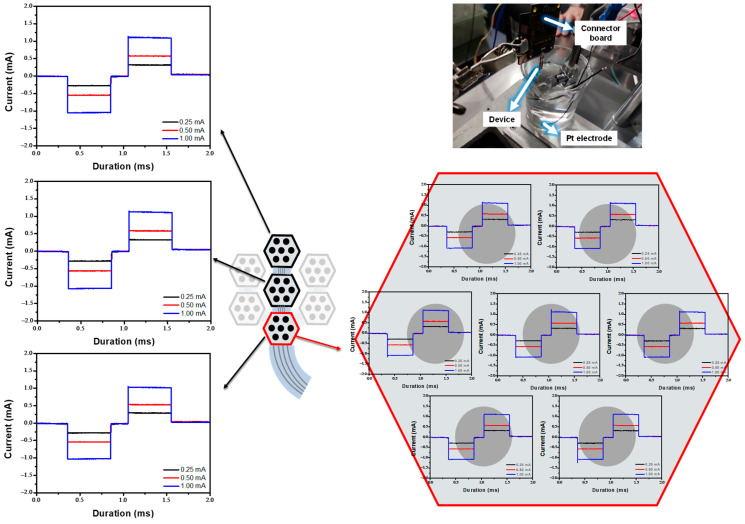
In vitro evaluation of the flexible MMRD in PBS solution.

To evaluate the device’s operation and performance, it was operated at various input currents to verify that the stimulating waveform and current value matched the designed input currents. To achieve charge-balanced neural stimulation, a cathodic-first biphasic current stimulation waveform was applied. The current response ranged from 250 to 1000 μA, with a pulse duration of 0.5 ms and an interpulse interval of 0.2 ms, as shown in Figure 6. Figure 6 shows an identical stimulation waveform with varying output currents among the three modules, as well as the respective stimulation waveforms for the seven electrodes within each module. Devices manufactured using the improved process operated the stimulation waveform successfully in PBS experiments. Minor overshoots of currents were observed during both positive and negative stimulation signal outputs. These overshoots are assumed to result from the release of the charge accumulated at the floating potential when switching the connection between the stimulation circuit and the counter electrode (in the floating control method). Since these overshoots are transient and involve a minimal amount of total charge, they are considered to have a negligible impact on biological tissues. These results indicated that the device, fabricated using the improved process, is suitable for the next stage of performance evaluation: in vivo experimentation.

### 3.2. EEP Signal Recording in the Rat Experiment

**Figure 7:**
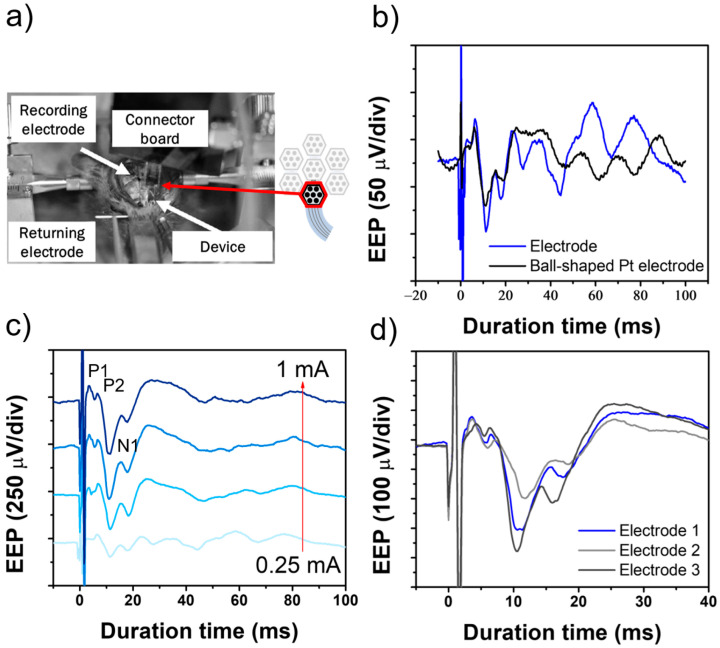
In vivo evaluation of the flexible MMRD in the rat experiment. (**a**) Setup of the EEP experiment. (**b**) Comparison of EEP signals using a ball-shaped electrode versus a fabricated device electrode. (**c**) EEP signals obtained with stimulation currents ranging from 0.25 to 1.0 mA. (**d**) EEP signals obtained with different electrodes at 1.0 mA stimulation current.

To assess whether the electrical stimulation capability of the device fabricated by the new process is sufficient to induce evoked potentials, we conducted in vivo experiments using rats. W electrodes were inserted into the SC to monitor changes in evoked potentials (resulting from retinal stimulation). A stimulation device placed on the sclera of the rat’s eyeball was used to stimulate the retina, and action potentials in the SC were recorded. The experimental setup for the EEP signal recording is presented in Figure 7a. Initially, the representative input stimulation waveforms were obtained with current settings of 0.25, 0.5, 0.75, and 1.0 mA. Control data were also collected using a Pt ball-shaped electrode for retinal stimulation (Figure 7b). Within milliseconds of retinal stimulation, we observed representative EEP responses, including the positive peak P1 and negative peak N1, approximately 5 and 10 ms after stimulation (Figure 7c). The timing and amplitude of these peaks are consistent with those reported in previous studies, demonstrating that the device developed in this study effectively induces neuronal responses. In addition, the EEP signals obtained from different electrodes were recorded, as shown in Figure 7d. The variations in EEP responses across electrodes suggest that neural activity induced by electrical stimulation is highly dependent on electrode positioning.

### 3.3. In Vivo Experiment in the Rabbit

**Figure 8:**
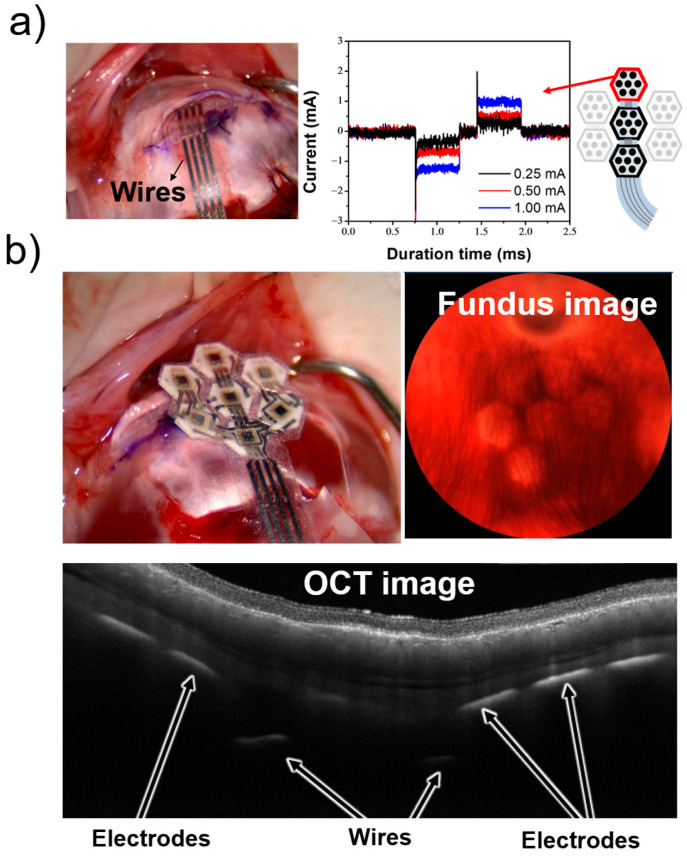
In vivo and in vitro evaluation of the device in the rabbit experiment. (**a**) Photograph of the rabbit eye with the implanted device and the stimulation waveform. (**b**) Obtained OCT and fundus images.

Next, we evaluated whether the flexible MMRD, embedded in the scleral pocket of a rabbit eye (which is approximately the same size as a human eye), performs with similar functionality as in rat experiments. Initially, we tested using a three-module device to assess the operation of multiple modules. In the rat experiments, the device was placed directly on the sclera, and stimulation was performed. In contrast, for the rabbit experiments, a scleral pocket was created, and the device was embedded in this pocket. To assess the electrical stimulation performance of implanted MMRDs in the rabbit scleral pocket, the corresponding biphasic cathodic-first stimulation waveforms were successfully observed across multiple Pt electrodes in the three-module retinal device. Representative stimulation waveform data obtained from retinal stimulation under these conditions are shown in Figure 8a. Similar to the rat experiments, the output from the device, with a stimulus current of up to 1.0 mA, closely resembled the set-current waveform. These results suggest that the device performs effectively even when embedded in the scleral pocket of the rabbit eye.

Following the aforementioned experiments and in preparation for future devices with a higher number of electrodes, we implanted a seven-module device into an extracted rabbit eye. The optical coherence tomography (OCT) and fundus images obtained during this procedure are shown in Figure 8b. These images indicate that the device can be successfully implanted in the desired position using the STS method. These experimental results demonstrate that the artificial retinal device developed in this study can effectively stimulate the retina (even when modularized). Furthermore, the STS method allows for precise implantation at the intended positions. All animal experiments were conducted in accordance with the protocols approved by NIDEK Co., Ltd.

## 4. Discussion

In previous studies, we outlined the fabrication process of a single-module STS retinal device [25,26]. However, issues with unreliable or disconnected wiring limited the development of multiple-module retinal devices. To address these issues, this study introduces an improved fabrication process incorporating multiple silane coupling modifications, an enhanced encapsulation process, and optimized sacrificial materials for device detachment to ensure reliable wiring. For instance, in our previous study, an acrylic substrate derived from liquid-type acrylic material was used as the sacrificial layer and fixation material for CMOS chip bonding. However, detaching the device from the acrylic substrate after CMOS chip installation proved to be challenging. In contrast, the PVA fixation method was employed in the flip-chip bonding process for CMOS chip installation, enabling a more reliable wiring process for multi-module retinal device fabrication due to its simpler detachment compared to acrylic substrates. Additionally, Au bumps mounted on the FPC provided more stable electrical connections to the Au/Ti wires through flip-chip bonding, outperforming conductive silver pastes. These process improvements have led to the successful fabrication of multi-module retinal devices. However, long-term implantation stability and biocompatibility remain a critical issue to be addressed. Although the device is encapsulated with biocompatible parylene-C, an established ion and water barrier material commonly used in implantable systems [28,29], comprehensive evaluation of its long-term durability and biocompatibility under physiological conditions is necessary. Such evaluation will be a primary focus in future studies. The in vivo experiments demonstrated that the fabricated artificial retinal device can effectively stimulate the retina even in its modularized configuration, and that implantation at the suprachoroidal location, as required by the STS method, is surgically feasible. While these experiments were conducted in healthy rats, future investigations will examine the device’s performance in retinal degeneration models such as rhodopsin Pro347Leu transgenic (Tg) rabbits. These studies will provide a more clinically relevant assessment of the device’s therapeutic potential. In summary, the device is capable of generating sufficient stimulation current for STS applications. Future work will focus on confirming whether the device can induce reliable electrical evoked potentials (EEPs) in retinal degeneration models and evaluate its long-term biostability in chronic implantation conditions.

Several research groups have measured EEPs in rats by electrically stimulating the retina [30,31,32,33,34,35]. While the amplitude of EEP responses reported in these studies starts from approximately 30 µV [35], the EEP responses obtained in this study are of greater magnitude and align with the timings of the response peaks observed in previous research. These findings suggest that the data obtained accurately represent EEPs generated by retinal stimulation and that electrical stimulation through the sclera effectively excites neurons in the SC, resulting in evoked potentials. In addition, retinal stimulation was performed using seven electrodes mounted on a ceramic substrate, and the resulting EEPs were measured. The variation in response waveforms from different stimulation electrodes indicates that different neuronal groups react according to the stimulation site. This experiment focused solely on measuring neural activity during stimulation using the fabricated artificial retinal device. Future research will benefit from incorporating fluorescence imaging alongside local field potential (LFP) recordings to compare stimulation electrode positions and neural response areas in greater detail. To achieve higher-precision vision, increasing the number of electrodes is crucial; however, because the implantation area is limited, maximizing the efficiency of each electrode’s stimulation performance is vital. Additionally, employing imaging methods that can cover broader areas than traditional electrophysiological techniques will enhance the ability to monitor whether retinal stimulation effectively promotes brain nerve activity.

## 5. Conclusions

This study focused on developing a fabrication process for a multi-module retinal device (MMRD) equipped with CMOS chips. While previous single-module devices faced issues with unreliable thin-film wiring, we introduced process improvements such as multiple silane coupling modifications, enhanced encapsulation, and the use of PVA for easier detachment in flip-chip bonding. Additionally, Au bumps on the flexible printed circuit (FPC) improved electrical connections with Au/Ti wiring. The fabricated MMRD successfully demonstrated the expected stimulation waveforms in both in vitro and in vivo environments, confirming its ability to stimulate the retina even in a modularized configuration. Furthermore, implantation using the STS method proved feasible, highlighting the potential of this approach for future retinal prostheses.

## Data Availability

Data are available upon request.

## Supplementary Materials

The following supporting information can be downloaded at: https://www.mdpi.com/article/10.3390/mi16050580/s1, Table S1: The chemical solution lists for etching the Au/Ti.

## Abbreviations

The following abbreviations are used in this manuscript:

CMOS	Complementary metal-oxide semiconductor
STS	Suprachoroidal transretinal stimulation
MMRD	Multi-module retinal devices
PaC	Parylene-C
EEP	Electrically evoked potential
OCT	Optical coherence tomography
LFT	Local field potential
