# Supplementary material for: Development and Evaluation of Multi-Module Retinal Devices for Artificial Vision Applications
Source: Micromachines (Basel). 2025 May 15;16(5):580. doi: 10.3390/mi16050580 (PMC12114173; doi:10.3390/mi16050580)
Supplement: Supplementary file 1 [file micromachines-16-00580-s001.zip › micromachines-3621774-supplementary.pdf]

Supporting Information

Development and Evaluation of Multi-Module Retinal Devices for Artificial Vision Applications

Kuang-Chih Tso [1,†], Yoshinori Sunaga [1,*,†], Yuki Nakanishi [2], Yasuo Terasawa [1,3], Makito Haruta [1,4], Kiyotaka Sasagawa [2,5] and Jun Ohta [1]

1 Institute for Research Initiatives, Nara Institute of Science and Technology, Ikoma 6300192, Japan
2 Division of Materials Science, Graduate School of Science and Technology, Nara Institute of Science and Technology, Ikoma 6300192, Japan
3 NIDEK Co., Ltd., Gamagori 4430036, Japan
4 Department of Opto-Electronic System Engineering, Faculty of Science and Engineering, Chitose Institute of Science and Technology, Chitose 0668655, Japan
5 Medilux Research Center, Graduate School of Science and Technology, Nara Institute of Science and Technology, Ikoma 6300192, Japan
* Correspondence: sunaga.yoshinori@ms.naist.jp
† These authors contributed equally to this work.

Table S1. The chemical solution lists for etching the Au/Ti.

	**Chemicals**
**Au etching solution**	125 g/L KI and 31.25 g/L I_2 aqueous solution
**Ti etching solution**	Pure Etch TE307<br>(Hayashi Pure Chemical Ind., Ltd., Osaka, Japan)
